# Effect of a 3% gelatin solution on urinary KIM-1 levels in patients after thyroidectomy: a preliminary randomized controlled trial

**DOI:** 10.1038/s41598-021-03108-y

**Published:** 2021-12-08

**Authors:** Patrycja Leśnik, Ewa Woźnica-Niesobska, Jarosław Janc, Magdalena Mierzchała-Pasierb, Lidia Łysenko

**Affiliations:** 1 Department of Anaesthesiology and Intensive Therapy, 4th Military Clinical Hospital, 50-560 Wrocław, Poland; 2 Department of Anaesthesiology and Intensive Therapy, Wroclaw Medical University, 50-981 Wrocław, Poland; 3 Department of Medical Biochemistry, Wroclaw Medical University, 50-369 Wrocław, Poland

**Keywords:** Endocrine system and metabolic diseases, Kidney diseases, Biomarkers, Diseases, Health care, Medical research, Nephrology, Risk factors, Signs and symptoms, Immunochemistry

## Abstract

Optimal fluid therapy significantly affects the maintenance of proper tissue perfusion and, consequently, kidney function. An adverse effect of colloids on kidney function is related to the incidence of postoperative kidney failure. The study aimed to assess the effect of a 3% gelatin solution on kidney function based on the urinary kidney injury molecule-1 (uKIM-1) level. This study used a parallel design and enrolled 64 adult patients with a mean age of 52.5 ± 13.1 years, all of whom underwent a thyroidectomy procedure under general anesthesia. Patients were randomly assigned to three comparison groups, each receiving a different dose of 3% gelatin solution during the thyroidectomy procedure. The patients from study groups A (n = 21) and B (n = 21) received a 3% gelatin solution at a dose of 30 ml/kg and 15 ml/kg body weight, respectively, during the first hour of the procedure. The patients from the control group C (n = 22) received an isotonic multi-electrolyte solution. Serum creatinine levels were determined, and urine samples were collected to determine levels of uKIM-1 before, 2 h, and 24 h after surgery. The patients’ demographic data, type and volume of fluid and hemodynamic status during the surgery were collected from relevant anesthesia protocols and were included in the study data. There were no statistically significant changes between groups in hemodynamic parameters such as systolic and diastolic blood pressure, heart rate, and oxygen saturation values. A statistically significant increase in uKIM-1 level was noted in patients receiving the 3% gelatin solution regardless of the dose. A statistically significant difference in uKIM-1 level was observed between groups A, B, and C measured 24 h after surgery, with the highest uKIM-1 level in group A. Measurement of uKIM-1 level could be an early and sensitive biomarker of kidney injury. Kidney toxicity of a 3% gelatin solution, evaluated based on the level of uKIM-1 in urine, correlates with transfused fluid volume. This study was retrospectively registered in the ISRCTN clinical trials registry (ISRCTN73266049, 08/04/2021: https://www.isrctn.com/ISRCTN73266049).

## Introduction

In recent years, much research has focused on the harmful effects of colloids used in patients hospitalized in the intensive care unit (ICU)^1,2^. Some colloids have been found to be associated with impaired kidney function, renal replacement therapy, and coagulation disorders and to lead to increased mortality^3,4^. However, only a few researchers have addressed the adverse effects of gelatin administered in the operating room^5^. Optimal fluid therapy in the perioperative setting is intended to maintain a euvolemic state and replenish the possible fluid losses associated with surgery. Optimal fluid therapy ensures the proper function of the circulatory system, optimal tissue perfusion, and kidney function. Maintaining a balanced fluid therapy is extremely difficult—the fluid type, volume, and rate of administration have to be adjusted for the type of surgery and its duration, preoperative fluid deficits, potential fluid losses, and co-morbidities of the patient. Numerous studies have established the adverse effects of colloids on kidney function and their relationship to the incidence of postoperative acute kidney injury (AKI)^6–8^. The development of kidney dysfunction in the perioperative setting leads to increased mortality, prolonged hospital stay, and higher costs of treatment^9,10^.

An early diagnosis of AKI is essential for effective treatment. Traditional blood and urine markers used to diagnose kidney injuries, such as urea and creatinine levels, are neither sensitive nor specific. In kidney injury, when the glomerular filtration rate (GFR) decreases, the half-life of creatinine increases in the period between 4 and 24–72 h after the GFR decreases. Therefore, a change in the creatinine level is delayed by up to 24 to 36 h. New biomarkers, such as neutrophil-gelatinase associated lipocalin (NGAL), cystatin C (CC), kidney injury molecule-1 (KIM-1), interleukin-18 (IL18), liver-type fatty acid-binding protein (L-FABP), N-acetyl-β-D-glucosaminidase (NAG), α-glutathione transferase (alpha-GST), metalloproteinase tissue inhibitor type 2 (TIMP-2) and insulin-like growth factor-binding protein 7 (IGFBP-7), might be more beneficial for early detection of acute kidney injuries^11^.

KIM-1 expression is low in healthy persons, and it is usually undetectable in urine, but its expression increases on the apical membrane of the nephron proximal tubule in the case of injury or ischemia. The level of KIM-1 is therefore markedly increased within hours following kidney injury^12^. KIM-1 has proven to be a specific marker of histopathological changes in the proximal tubule in response to ischemia, hypoperfusion, or toxicants. It is expressed to a lesser extent in the case of an infection or chronic kidney disease^13,14^. After the injury, the extracellular domains of KIM-1 separate from the cell surface and enter the urine^15^. Previous studies have shown that tissue expression and urinary excretion of KIM-1 are sensitive and specific markers of AKI^16,17^.

Colloids are fluids used primarily for a hemorrhagic shock when the rapid securing of blood products is not possible^18–20^. Gelatin preparations can be extremely useful in these situations, but establishing their adverse effects on kidney function is essential^21,22^. Therefore, this study aimed to determine the effect of the gelatin solution used intraoperatively during a thyroidectomy on kidney function by assessing the urinary KIM-1 (uKIM-1) level. In addition, we hypothesized that the nephrotoxicity of gelatin solutions depends on the dose and total volume of transfused fluid and that gelatin solutions might have a nephrotoxic effect in patients with coexisting renal disease regardless of dose.

## Results

**Figure 1:**
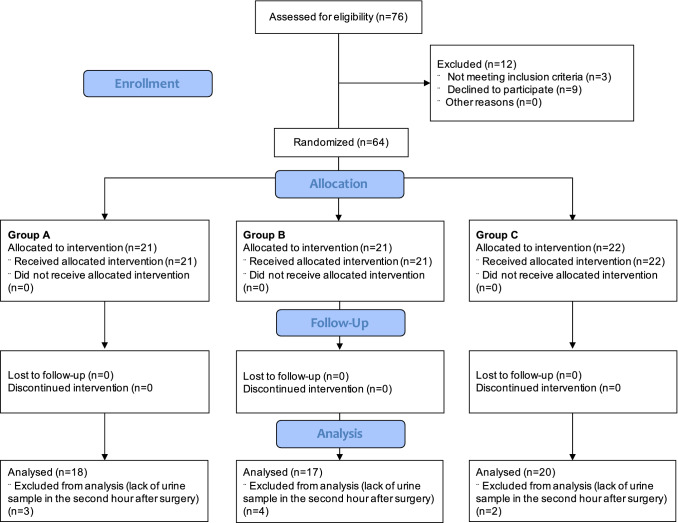
CONSORT flow chart of patients included in the study.

In the analyzed period, 126 patients, including 97 females and 29 males, qualified for a thyroidectomy procedure. Of these, 21 patients (15 females and 6 males) did not consent to participate in the study. Thus, a group of 105 patients (82 females and 23 males) were enrolled in the study. Forty-one patients (25 females and 16 males) were excluded due to an American Society of Anaesthesiologists (ASA) score > II. Ultimately, 64 patients (57 females and 7 males) with an ASA score of I–II were enrolled in the study. Another nine patients (2 females and 7 males) were excluded during further observation due to study protocol violation (no urine sample 2 h after the procedure). The Consolidated Standards of Reporting Trials (CONSORT) 2010 flow diagram for the study process is presented in Fig. 1.

Ultimately, the analysis involved 55 patients, all of whom were female (Table 1). In the analyzed period, two types of infusion fluids were routinely used intraoperatively: a 3% gelatin solution (Geloplasma, Fresenius Kabi, Warsaw, Poland) and a balanced crystalloid solution (Multi-Electrolyte Fluid, Fresenius Kabi, Warsaw, Poland). On the day of surgery, patients were randomly assigned into one of three comparative groups: A, B, and C. Groups A and B included patients who received the 3% gelatin solution at a dose of > 20 mL/kg and ≤ 20 mL/kg of body mass, respectively, while group C included patients who received a balanced crystalloid solution. Ultimately, out of 55 patients, 18 were assigned to group A (mean dose of 3% gelatin solution 23.3 mL/kg), 17 patients were assigned to group B (mean dose of 3% gelatin solution 15 mL/kg), and 20 patients were assigned to group C (mean dose of balanced crystalloid solution 27 mL/kg).

**Table 1:** Baseline demographic data.

Item	Total (n = 55) M ± SD	Group A (n = 18) Me (Q1; Q3)	Group B (n = 17) Me (Q1; Q3)	Group C (n = 20) Me (Q1; Q3)	*p*
Age (years)	52.5 ± 13.1	53 (46; 64)	53 (42; 59)	51 (37; 65)	0.692
Weight (kg)	73.0 ± 11.1	75 (65; 81)	79 (70; 90)	69 (63; 75)	0.022*
Height (cm)	163.7 ± 5.8	164 (160; 65)	165 (163; 170)	162 (157; 168)	0.157
BMI (kg/m2)	27.3 ± 4.1	28.1 (24.5; 30.4)	27.7 (24.8; 29.7)	27.3 (23.6; 28.6)	0.223
BSCL (mg/dL)	0.78 ± 0.11	0.75 (0.70; 0.79)	0.83 (0.74; 0.84)	0.76 (0.71; 0.87)	0.463

*BMI* body mass index, *BSCL* baseline serum creatinine level, *M* mean, *SD* standard deviation, *Me* median, *Q1* quartile 1st, *Q3* quartile 3rd, *n* number of participants, *p* level of statistical significance.

Apart from the statistically significant difference in mean body weight between groups B and C (78.7 kg and 68.8 kg, respectively, *p* = 0.023), we did not find any significant difference between the groups regarding age, height, or BMI. Furthermore, the mean surgery time was 81.0 ± 25.0 min for the whole population, with no statistically significant difference between the analyzed groups (for groups A, B, and C, the times were 88 [70 ÷ 105], 75 [65 ÷ 90], and 70 [60 ÷ 90] minutes, respectively; *p* = 0.393).

In the first hour of the surgery, patients in group A received 23.3 (19.7 ÷ 25.0) mL/kg of the 3% gelatin solution, group B received 15.0 (12.8 ÷ 16.7) mL/kg of the 3% gelatin solution, and group C received 27.1 (24.2 ÷ 2.5) mL/kg of the Multi-Electrolyte Fluid (*p* < 0.001). In the second hour of the surgery, the patients received 11.9 (2.7 ÷ 14.5), 14.0 (12.4 ÷ 15.4), and 3.0 (0.0 ÷ 11.9) mL/kg in groups A, B, and C, respectively. In this second hour of the surgery, patients in group B received a statistically higher dose of fluid than patients in groups A and C (*p* < 0.001).

### Hemodynamic parameters

**Figure 2:**
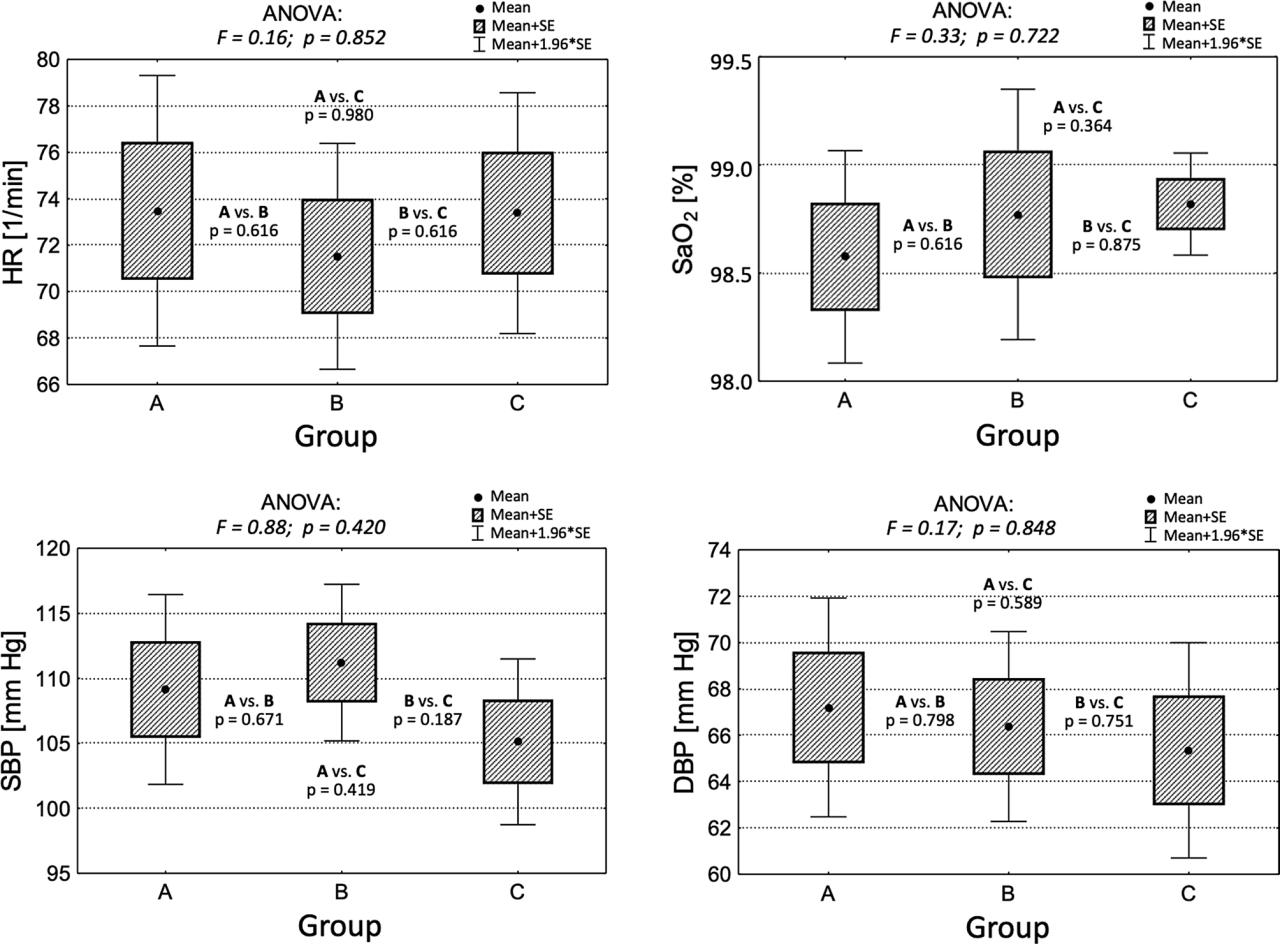
Hemodynamic parameters in groups A, B, and C.

There were no statistically significant differences in patients’ hemodynamic statuses between any of the groups (Table 2). In addition, there were no statistically significant changes between groups A, B, and C in hemodynamic parameters such as systolic and diastolic blood pressure, heart rate, and oxygen saturation values (Fig. 2). During surgery, hemodynamic parameters remained stable, regardless of the fluid therapy regimen used. None of the patients required the use of pressor amines. Bleeding requiring transfusion of blood preparations occurred in none of the analyzed cases.

**Table 2:** Hemodynamic status of the patients during the surgery.

	Total (n = 55) M ± SD	Group A (n = 18) Me (Q1; Q3)	Group B (n = 17) Me (Q1; Q3)	Group C (n = 20) Me (Q1; Q3)	*p*
HR (min^−1^)	72.6 ± 11.4	69 (65; 79)	71 (63; 80)	71 (66; 80)	0.852
SBP (mmHg)	108.2 ± 14.1	109 (105; 118)	110 (102; 120)	105 (100; 114)	0.420
DBP (mmHg)	66.2 ± 9.6	65 (60; 71)	66 (62; 73)	63 (58; 70)	0.848
SaO_2_ (%)	98.7 ± 0.9	99 (98; 99)	99 (98; 100)	99 (99; 99)	0.722

*HR* heart rate, *SBP* systolic blood pressure, *DBP* diastolic blood pressure, *SaO*_*2*_ arterial blood saturation, *M* mean, *SD* standard deviation, *Me* median, *Q1* quartile 1st, *Q3* quartile 3rd, *n* number of participants, *p* level of statistical significance.

### Serum creatinine

**Figure 3:**
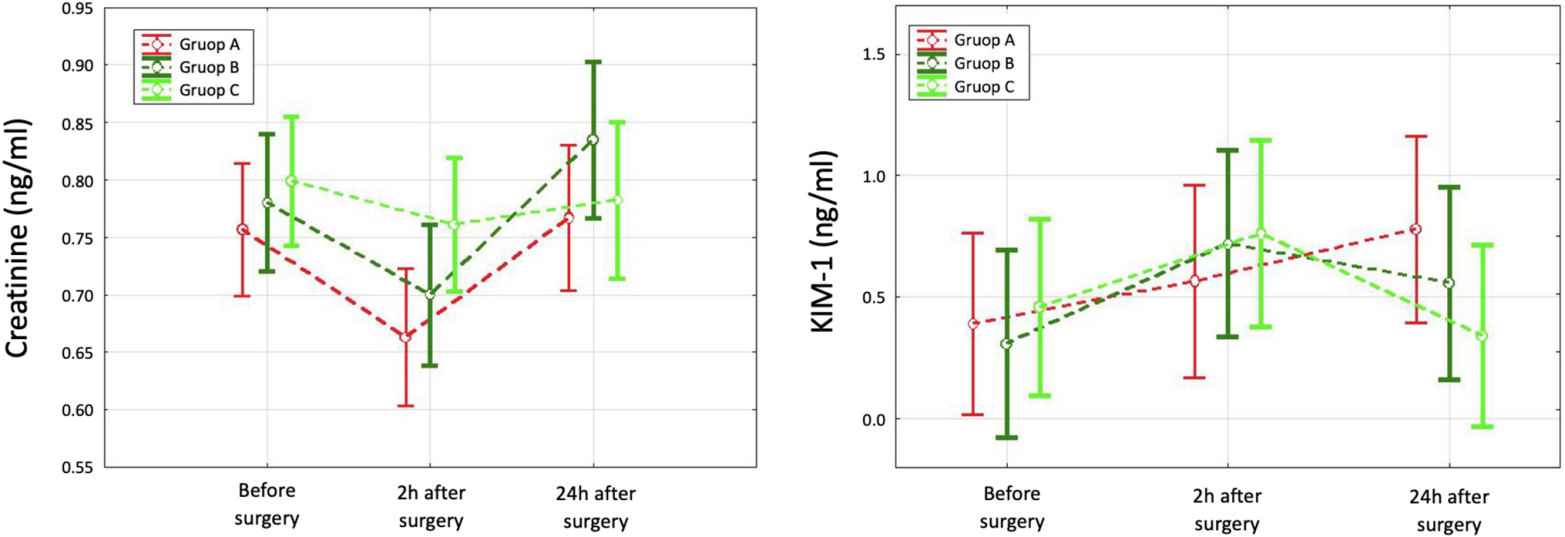
Creatinine and KIM-1 levels before and 2 h and 24 h after surgery.

The serum creatinine levels in the preoperative and postoperative periods were within the normal range in all patients (0.8–1.3 mg/dL). There was no statistically significant difference between the groups in creatinine levels in the baseline (Table 1), 2 h after surgery (0.69 [0.62 ÷ 0.75], 0.74 [0.62 ÷ 0.83] and 0.70 [0.65 ÷ 0.80] for groups A, B and C, respectively; *p* = 0.71) and 24 h after surgery (0.75 [0.71 ÷ 0.82], 0.85 [0.78 ÷ 0.91] and 0.78 [0.69 ÷ 0.82] for groups A, B and C respectively; *p* = 0.068). See Fig. 3.

In the whole analyzed population, serum creatinine level 2 h after surgery was statistically significantly lower than it was both before surgery (0.71 [0.64 ÷ 0.80] vs. 0.76 [0.70 ÷ 0.85]; *p* < 0.001) and 24 h after surgery (0.71 [0.64 ÷ 0.80] vs. 0.78 [0.72 ÷ 0.85]; *p* < 0.001). There was no statistically significant difference between baseline serum creatinine and serum creatinine levels 24 h after surgery (*p* = 0.672). Among the analyzed groups, a statistically significant decrease in serum creatinine levels 2 h after surgery, as compared to the baseline, was observed in group A (*p* = 0.001) and group C (*p* = 0.014). In comparison, a statistically significant decrease in serum creatinine 2 h after surgery, as compared to 24 h after surgery, was observed in groups A (*p* = 0.014) and B (*p* = 0.007).

### Urinary kidney injury molecule

**Figure 4:**
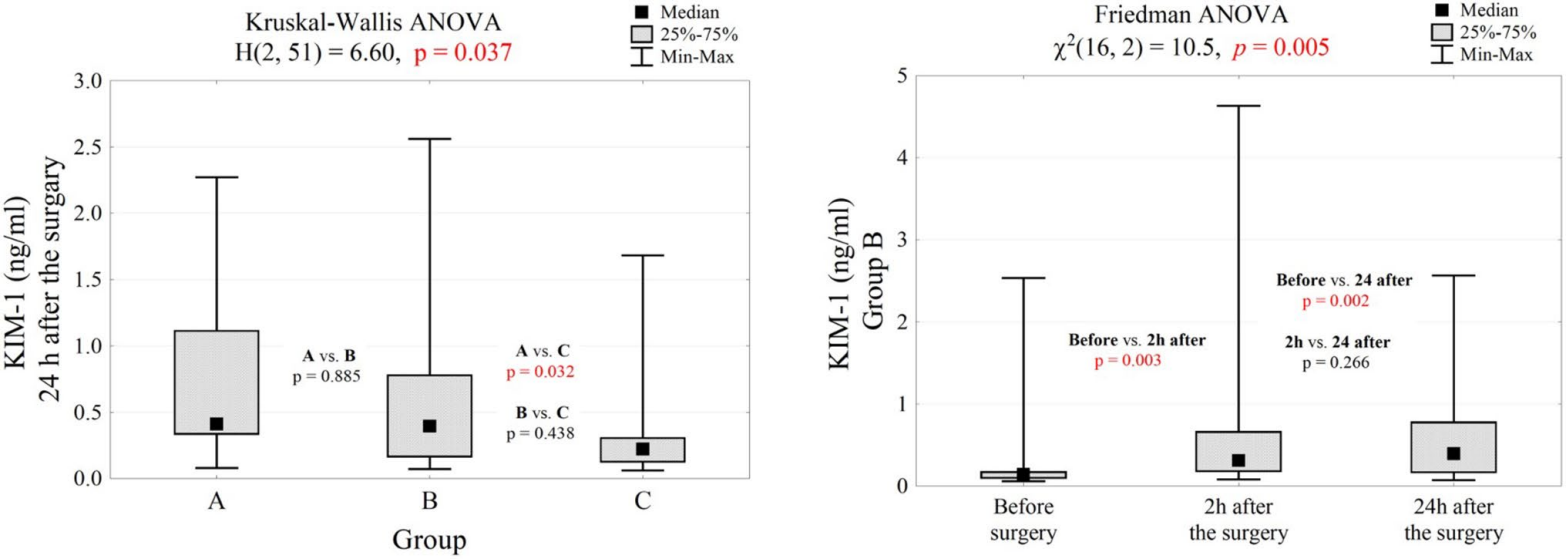
KIM-1 levels in groups A, B, and C measured 24 h after surgery, and in group B measured before and 2 h and 24 h after surgery.

There was no statistically significant difference between groups A, B, and C in the uKIM-1 level before and 2 h after surgery. However, a statistically significant difference in uKIM-1 level was observed between groups A, B, and C 24 h after surgery, with the highest uKIM-1 level in group A, where the highest volume of the 3% gelatin solution was administered (Table 3 and Figs. 3 and 4).

**Table 3:** uKIM-1 levels before the surgery, and 2 h and 24 h after surgery.

uKIM-1 (ng/ml)	Total M ± SD	Group A Me (Q1; Q3)	Group B Me (Q1; Q3)	Group C Me (Q1; Q3)	*p*
Before surgery	n = 54 0.39 ± 0.54	n = 18 0.20 (0.12; 0.35)	n = 17 0.14 (0.06; 0.18)	n = 19 0.20 (0.13; 0.59)	0.111
2 h after surgery	n = 50 0.68 ± 1.13	n = 16 0.38 (0.23; 0.66)	n = 17 0.31 (0.17; 0.67)	n = 17 0.31 (0.20; 0.48)	0.727
24 h after surgery	n = 51 0.55 ± 0.60	n = 17 0.41 (0.33; 1.12)	n = 16 0.40 (0.16; 0.79)	n = 18 0.22 (0.12; 0.31)	0.037*
Friedman ANOVA		*p* = 0.066	*p* = 0.005	*p* = 0.646	×

*uKIM-1* the urinary kidney injury molecule-1, *M* mean, *SD* standard deviation, *Me* median, *Q1* quartile 1st, *Q3* quartile 3rd, *n* number of participants, *p* level of statistical significance.

A statistically significant increase in uKIM-1 level 2 h after surgery, as compared to the baseline, was observed only in group B (0.1 [0.1 ÷ 0.2] vs. 0.3 [0.2 ÷ 0.7]; *p* = 0.003). A statistically significant increase in uKIM-1 levels 24 h after surgery, as compared to the baseline, was observed in groups A (0.2 [0.1 ÷ 0.4] vs. 0.4 [0.3 ÷ 1.1]; *p* = 0.039) and B (0.1 [0.1 ÷ 0.2] vs*.* 0.4 [0.2 ÷ 0.8]; *p* = 0.002). A statistically significant change in uKIM-1 level was observed between groups A and C (0.78 vs. 0.34, respectively; *p* = 0.032) 24 h after surgery.

## Discussion

It is well known that both the type of fluid and the amount of fluid administered impact kidney function in the perioperative period^23^. However, our study was the first to evaluate the effects of the gelatin solution infusion on kidney function based on the uKIM-1 level in the perioperative period of non-cardiac surgery.

The results of our study confirm a potentially toxic effect of the gelatin solution on kidney function. In both groups receiving the 3% gelatin infusion, a significant increase in uKIM-1 levels was noted. In addition, a comparison of the groups receiving different types of fluids led to the hypothesis that both fluid type and dose affect kidney function^24^.

The patients who qualified for our study were those undergoing surgery with a potentially low risk of intraoperative bleeding. AKI develops in 7.5% of patients in the postoperative period after non-cardiac surgery and is associated with an eight-fold increase in mortality within 30 days of surgery^25,26^. One of the main causes of postoperative renal failure is intraoperative hypotension, particularly when a mean arterial pressure (MAP) is < 55 mm/Hg and lasts more than 10 min^27^. According to the KDIGO (Kidney Disease: Improving Global Outcomes) guidelines^28^, major surgery is among the risk factors for the development of AKI. In order to eliminate additional factors affecting renal function in this study, procedures of low invasiveness were selected, and arterial blood pressure was strictly controlled during surgery, without hypotension observed. Additionally, all patients underwent a uniform anesthetic regimen with propofol, fentanyl, and rocuronium, in doses adjusted to body weight—this enabled assessment of the effects of the fluids used on renal function by evaluating uKIM-1 concentration.

The hemodynamic status of our patients was stable, and proper tissue perfusion was obtained; this allowed for an assumption that the fluid balance was adequately maintained and allowed hypoperfusion periods to be ruled out as a potential cause of kidney function impairment. Also, decreased serum creatinine levels and increased GFR values in the second hour after surgery confirmed that an optimal fluid balance was most likely maintained.

Both an increase in serum creatinine level and impairment of diuresis are symptoms of already developed kidney dysfunction and provide AKI definition elements according to Acute Kidney Injury Network (AKIN) criteria^29^. There was no increase in serum creatinine levels within 24 h after surgery in our study population. Therefore, diuresis volume was not determined, but none of the patients reported any diuresis issues following surgery. As we could not show any adverse effects of the fluids administered on kidney function using the AKIN definition, we decided to analyze kidney function using an alternative biomarker: uKIM-1.

An increase in uKIM-1 level precedes increased creatinine levels and a decrease in diuresis, allowing for the faster implementation of prophylactic measures to prevent overt AKI development. In addition, the uKIM-1 level is described as an early and sensitive biomarker of kidney tubule injury^12,30^.

An experimental study by Boy et al.^31^ on the canine shock model showed the harmful effect of gelatin on kidney function, as it increases uKIM-1 levels. The same hypothesis was also confirmed in our study. Both groups (group A and group B) receiving the gelatin solution infusion increased the uKIM-1 levels significantly. Moreover, the administered fluid volume (3% gelatin solution) also affected kidney function. The highest uKIM-1 level 24 h after surgery was observed in group A, where the highest volume of the 3% gelatin solution had been administered (23.3 [19.7 ÷ 25.0] mL/kg). The results are consistent with the available literature reports^32^.

Our analysis of uKIM-1 as a biomarker showed that, in comparison to creatinine, it could be a sensitive and specific kidney function marker. The results are consistent with the Food and Drug Administration (FDA) data that includes uKIM-1 as a useful marker of drug-induced kidney injury^33^. Even though uKIM-1 is not present in healthy volunteers’ urine^34^, our results show that only levels ≥ 0.32 ng/mL can predict kidney injury.

### Limitations of the study

This study has some potential methodological limitations to be mentioned. First, this was a single-center study; thus, the generalizability of the results to the whole country is insufficient. Well-designed multi-center studies are needed. However, it should be pointed out that this study has been designed as a preliminary randomized controlled trial (RCT). In addition, despite adequate sample size calculation, the number of patients included in the study was relatively small, and the study sample should be larger in the future to avoid undermining the statistical power of the test and minimize the formation of artifacts. Notably, only female patients were included in the analysis period, so the results only refer to the female gender and should not be related to male patients. Future studies should include examinations performed on patients of both sexes and consider any inter-gender differences in uKIM-1 levels. In addition, none of the patients included in the study developed overt AKI; therefore, it would be worthwhile to design studies that also include patients with developed AKI in the context of evaluating the therapeutic effect of a 3% gelatin solution on renal function based on the uKIM-1 level as a specific marker of kidney tissue injury.

### Further research directions

An early diagnosis of kidney damage allows for rapid implementation of treatment or elimination of the damaging factor. For this purpose, it is necessary to use biomarkers with high specificity and sensitivity. The search for such a marker should be a priority in view of the increasing number of drugs and therapeutic substances with nephrotoxic effects.

## Conclusions

The uKIM-1 level could be an early and sensitive biomarker of kidney injury. Determination of uKIM-1 levels in patients undergoing thyroidectomy has shown that intraoperatively administered gelatin solutions can have detrimental dose-dependent effects on the kidneys. A 3% gelatin solution at a dose over 15 ml/kg body weight has been shown to have nephrotoxic effects.

## Methods

### Participants

The study was designed as a prospective parallel randomized clinical trial. The study was conducted between April 2014 and December 2014 at the Department of Anaesthesiology and Intensive Care and the Department of Surgery at the University Hospital in Wroclaw, Poland. The study initially recruited 126 patients, of which 76 were assessed for eligibility. A group of 12 patients did not fulfill the below eligibility criteria (n = 3) or declined to participate (n = 9) and were excluded from further stages of the study. Ultimately, the study sample included 64 patients with a mean age of 52.5 ± 13.1 years. No patients were lost to follow-up or did not receive the allocated intervention. It should be noted that 9 patients (n = 3 in group A, n = 4 in group B, and n = 2 in group C) were excluded from analysis due to a lack of urine samples in the second hour after surgery (Fig. 1).

### Enrolment

The study enrolled consecutive adult patients, irrespective of gender, with an ASA I or II, who underwent partial or complete thyroid gland removal in the analyzed period as well as who had no contraindications for the administration of a 3% gelatin solution (no gelatin allergy), no blood coagulation disorders and no allergy to anesthetic agents. Informed and written consent to participate in the study was obtained. The exclusion criteria comprised an ASA score > II, impaired kidney function before the surgery (serum creatinine > 1.3 mg/dL), current immunosuppressive therapy, confirmed gelatin allergy or anesthetics allergy, blood coagulation disorders, or refusal to participate in the study. The patients’ enrolment was performed by researcher Patrycja Leśnik.

### Randomization

On the day of surgery, all patients were randomly assigned into one of three comparative groups: A, B, and C. Allocation concealment was provided by the researcher Patrycja Leśnik using central randomization in a 1:1:1 ratio generated by computer software (https://www.random.org). Before the study, a list of patients with their respective numbers and group assignments was generated. The randomization list was given to the anesthesiologist by the coordinating nurse on the day of surgery in the operating theatre. A triple blinding procedure was used that involved participants, as well as the assessor and data analyst.

### Ethics

The study protocol was approved by the Bioethics Committee of the Wroclaw Medical University in Poland (permission no. KB–283/2013). The study was carried out in accordance with the guidelines of the Declaration of Helsinki and Good Clinical Practice. Informed and written consent was obtained from all the patients. During the premedication visit on the day preceding the surgery, the patients gave their written informed consent to participate in the study. The standards from CONSORT were followed, and the CONSORT checklist was used for enrolment and allocation of patients^35^. The study was retrospectively registered in the ISRCTN clinical trials registry with reference number ISRCTN73266049 on 8 April, 2021. The study protocol is available in the ISRCTN registry at https://www.isrctn.com/ISRCTN73266049.

### General anesthesia

Patients received one peripheral intravenous access in the operating theatre, 5-lead electrocardiogram (ECG), non-invasive blood pressure (NIBP), and pulse oximeter. Following the Clinic of General, Gastroenterological, and Endocrine Surgery standard procedure, patients had their last meal in the afternoon of the day before surgery and received fluids until midnight. The same standard general anesthesia procedures were used in all anesthetized patients. Anesthesia was induced with propofol 2 mg/kg body weight, fentanyl 0.1 mg, and rocuronium 0.6 mg/kg body weight, followed by maintenance of anesthesia with inhaled sevoflurane and intravenous fentanyl. During surgery, arterial blood NIBP was monitored at 5-min intervals, and continuous ECG, SaO2, and train-of-four (TOF) monitoring was used.

### Measures

The primary study outcome was to observe changes in uKIM-1 levels determined from urine samples using an enzyme-linked immunoassay (ELISA) method. The secondary study outcome was to detect serum creatinine level changes determined from venous blood samples using laboratory methods. The same measurement timepoints were set for both outcomes at baseline, postoperative 2 h, and postoperative 24 h. Demographic data (age, weight, height) were included in the study data.

#### Hemodynamic parameters

Heart rate (HR, measured continuously), arterial blood oxygen saturation (SaO_2_, measured continuously), systolic (SBP), and diastolic blood pressure (DBP, measured every 5 min), as well as data concerning the type and volume of fluids administered during the first and second hour of the procedure, were collected from relevant anesthesia protocols and included in the study data.

#### Blood pressure measurements

Measurements were performed by the auscultatory method using a certified mercury sphygmomanometer in the operating room during surgery. The patient’s arm was bare and placed at heart level, and a standard cuff was used and fitted appropriately to the subject’s arm size. Both parameters SBP and DBP, were recorded every 5 min.

#### Cardiac electrical activity measurements

Electrical activity going through the heart was measured by skin electrodes. In addition, the 3-channel electrocardiogram was used. Red is on the right arm, yellow on the left arm, green on the left leg (‘sun shines on the grass’), and black on the right leg. These basic leads yield enough information for rhythm monitoring.

#### Blood saturation measurements

Pulse oximeter measurements were performed by the sensor device, which was placed on a fingertip. The device passes two wavelengths of light through the body part to a photodetector. It measures the changing absorbance at each wavelength, allowing it to determine the absorbances due to pulsing arterial blood.

#### Serum samples analysis

Serum creatinine level was determined in blood samples collected via venous cannula before surgery and 2 h and 24 h after surgery. The determination of creatinine in serum samples was performed with an enzymatic method through the Alinity c Creatinine Reagent Kit (Abbott Laboratories, Irving, United States) and the analyzer Alinity (Abbott Laboratories, Irving, United States). The limit of detection for serum creatinine was 0.05 mg/dl (4.5 μmol/l).

#### Urinary samples analysis

Urine samples of 15 mL were collected from patients by spontaneous micturition on the day preceding the surgery and then 2 h and 24 h after surgery to determine KIM-1 level. The 15 mL urine samples were collected in urinary cups and centrifuged at 2000–3000 rpm for 20 min; the supernatant obtained was stored at − 72 °C. The quantitative determination of uKIM-1 was performed using an enzyme-linked immunoassay (ELISA) kit according to the manufacturer’s protocol: Human Urinary TIM-1/KIM-1/HAVCR Quantikine ELISA Kit (R&D Systems, Minneapolis, USA). The test was performed on a 96-well plate coated with an anti-human KIM-1 antibody. At first, 100 µl of assay diluent was added to each well. Then, 50 µl of the standard at concentrations of 0, 0.156, 0.312, 0.625, 1.25, 2.5, 5 and 10 ng/ml was added in duplicate. The remaining wells were filled with 50 µl of patient and control urine, added in duplicate. After 2 h of incubation at room temperature, the liquid from each well was removed. The wells were washed four times with 400 µl of a wash buffer. Then, 200 µl of human TIM-1 conjugate was added to each well and left for 2 h at room temperature. After 2 h of incubation, the wells were washed four times with 400 µl of a wash buffer. Subsequently, 200 µl of substrate solution was added to each well, and it was left for 30 min at room temperature in the dark. After 30 min of incubation at room temperature, the reaction was stopped with a stop solution. Absorbance was read using a microplate reader, the Tecan Infinite 200 (Tecan Austria GmbH, Salzburg, Austria), at 450 nm and 540 nm (wavelength correction). The minimum detectable level of human KIM-1 ranged between 0.003 ÷ 0.046 ng/ml.

### Procedure

The person performing the below-mentioned experimental procedures was the same anaesthesiologist for all groups, who had at least 5 years of professional expertise. The primary general purpose of the study was prevention in terms of assuring proper kidney function and preventing kidney damage. Therefore, all patients were carefully monitored for possible adverse events such as allergic reactions to local anesthetics, technical difficulties of the blockade, or hematoma.

The patients from the first group (A, an experimental group) received an anaesthesiologic intervention in the form of a 3% gelatin solution (Geloplasma, Fresenius Kabi, Warsaw, Poland) at a dose of 30 ml/kg body weight during the first hour of the procedure and then the isotonic multi-electrolyte solution (Fresenius Kabi, Warsaw, Poland) at a dose of 15 ml/kg body weight/hour.

The patients from the second group (B, an experimental group) received an anesthesiologic intervention in the form of a 3% gelatin solution (Geloplasma, Fresenius Kabi, Warsaw, Poland) at a dose of 15 ml/kg body weight during the first hour of the procedure and then the isotonic multi-electrolyte solution (Fresenius Kabi, Warsaw, Poland) at a dose of 15 ml/kg body weight/hour.

The patients from the third group (C, the control group) received an active-controlled intervention in the form of an isotonic multi-electrolyte solution (Fresenius Kabi, Warsaw, Poland) at a dose of 30 ml/kg body weight during the first hour of the procedure and then the isotonic multi-electrolyte solution (Fresenius Kabi, Poland) at a dose of 15 ml/kg body weight/hour.

### Sample size

For the analysis of variance (ANOVA) with three groups and three repeated measures, the sample size of at least N = 30 was needed for detecting a large effect size (Cohen’s *f* = 0.5) with the statistical power of 80% and α = 0.05. For one-way ANOVA with three groups, the sample size of at least N = 42 was needed for detecting a large enough effect size (Cohen’s *f* = 0.5) with a statistical power of 80% and α = 0.05. Statistical power analysis was conducted with the software G*Power version 3.1.9 (Heinrich-Heine-Universität Düsseldorf, Germany).

### Statistical analysis

Statistical analysis was performed using Statistica software version 13.3 (TIBCO Software Inc., Palo Alto, California, United States). The distribution of all the quantitative parameters (age, body weight, height, BMI, uKIM-1 level) was checked for consistency with the normal distribution. The conformity assessment was carried out using the Shapiro–Wilk and Kolmogorov–Smirnov tests. The critical significance level was assumed at *p* < 0.05. The significance of the differences in the mean values between two groups (e.g., baseline level to 2-h level) for normal distribution parameters and homogeneous variances was checked with the Student’s t-test for the related variables. The significance of differences in average values (medians) between two groups for parameters with a distribution considerably deviating from the theoretical normal distribution or heterogeneous variances was verified with the Wilcoxon test. For variables that were not significantly different from the normal distribution, ANOVA was used. The Tukey’s honest significant difference (HSD) test was used as a post-hoc test; thus, the observed significance level included performing multiple comparisons. For variables that were significantly different from the normal distribution, the Kruskal–Wallis test was used, and for dependent variables, the Friedman test was used. The Dunn’s test was used as a post-hoc test, corrected for multiple testing (the Bonferroni correction was used here). The results were considered statistically significant when the *p*-values were lower than 0.05.

## Data Availability

The datasets generated during and/or analyzed during the current study are available from the corresponding author on reasonable request.
